# Nuclear Factor kappa B is central to Marek’s Disease herpesvirus induced neoplastic transformation of CD30 expressing lymphocytes in-vivo

**DOI:** 10.1186/1752-0509-6-123

**Published:** 2012-09-14

**Authors:** Shyamesh Kumar, Dusan Kunec, Joram J Buza, Hsin-I Chiang, Huaijun Zhou, Sugalesini Subramaniam, Ken Pendarvis, Hans H Cheng, Shane C Burgess

**Affiliations:** 1 Department of Pathobiology and Population Medicine, Mississippi State University, PO Box 6100, MS, Mississippi State, 39762, USA; 2 Institut für Virologie, Freie Universität Berlin, Berlin, Germany; 3 School of Life Sciences and Bioengineering, Nelson Mandela African Institute of Science and Technology, PO Box 447, Arusha, Tanzania; 4 Department of Bioengineering, University of California-San Diego, 9500 Gilman Dr, La Jolla, CA, 92093, USA; 5 Department of Poultry Science, College of Agriculture and Life Sciences, Texas A&M University, College Station, TX, 77843, USA; 6 Comparative Medicine and Integrative Biology Graduate Program, College of Veterinary Medicine, Michigan State University, East Lansing, MI, 48824, USA; 7 College of Agriculture and Life Sciences, University of Arizona, P.O. Box 210036, Tucson, AZ, 85721, USA; 8 USDA-ARS, Avian Disease and Oncology Laboratory, 4279 East Mount Hope Road, East Lansing, MI, 48823, USA

**Keywords:** Marek’s disease, Lymphomas, Meq, NF-κB, Genetic resistance, CD30, Proteomics

## Abstract

**Background:**

Marek’s Disease (MD) is a hyperproliferative, lymphomatous, neoplastic disease of chickens caused by the oncogenic *Gallid herpesvirus type 2* (GaHV-2; MDV). Like several human lymphomas the neoplastic MD lymphoma cells overexpress the CD30 antigen (CD30^hi^) and are in minority, while the non-neoplastic cells (CD30^lo^) form the majority of population. MD is a unique natural in-vivo model of human CD30^hi^ lymphomas with both natural CD30^hi^ lymphomagenesis and spontaneous regression. The exact mechanism of neoplastic transformation from CD30^lo^ expressing phenotype to CD30^hi^ expressing neoplastic phenotype is unknown. Here, using microarray, proteomics and Systems Biology modeling; we compare the global gene expression of CD30^lo^ and CD30^hi^ cells to identify key pathways of neoplastic transformation. We propose and test a specific mechanism of neoplastic transformation, and genetic resistance, involving the MDV oncogene Meq, host gene products of the Nuclear Factor Kappa B (NF-κB) family and CD30; we also identify a novel Meq protein interactome.

**Results:**

Our results show that a) CD30^lo^ lymphocytes are pre-neoplastic precursors and not merely reactive lymphocytes; b) multiple transformation mechanisms exist and are potentially controlled by Meq; c) Meq can drive a feed-forward cycle that induces CD30 transcription, increases CD30 signaling which activates NF-κB, and, in turn, increases Meq transcription; d) Meq transcriptional repression or activation of the CD30 promoter generally correlates with polymorphisms in the CD30 promoter distinguishing MD-lymphoma resistant and susceptible chicken genotypes e) MDV oncoprotein Meq interacts with proteins involved in physiological processes central to lymphomagenesis.

**Conclusions:**

In the context of the MD lymphoma microenvironment (and potentially in other CD30^hi^ lymphomas as well), our results show that the neoplastic transformation is a continuum and the non-neoplastic cells are actually pre-neoplastic precursor cells and not merely immune bystanders. We also show that NF-κB is a central player in MDV induced neoplastic transformation of CD30-expressing lymphocytes in vivo. Our results provide insights into molecular mechanisms of neoplastic transformation in MD specifically and also herpesvirus induced lymphoma in general.

## Background

Lymphomas are the 6^th^ leading cause of cancer mortality in the USA especially in patients younger than 40 years [1,2]. More than 11% of human lymphomas overexpress the CD30 antigen (a.k.a. Hodgkin’s Disease antigen, or Tumor Necrosis Factor Receptor Superfamily Member 8 [TNFRSF8])—this includes all Hodgkin’s lymphomas (HL) and some non-Hodgkin’s lymphomas (NHL); e.g. anaplastic large cell lymphoma (ALCL), primary cutaneous anaplastic large cell lymphoma (PCTL), adult T-cell leukemia/lymphoma (ATLL), peripheral T-cell lymphoma (PTCL), natural killer (NK)/T-cell lymphoma, nasal and enteropathy type T cell lymphoma [3-5]. Natural “spontaneous” animal models that mimic the human lymphoma microenvironment, and have a functional immune system, are invaluable tools to understand lymphoma development [6]. Marek’s Disease (MD)—a CD4+ T cell lymphoma of chickens caused by the *Gallid herpesvirus type 2* (GaHV-2; MDV)—is a unique natural animal model for herpesvirus induced lymphomagenesis in general and CD30^hi^ lymphomas specifically [7].

CD30 overexpression is an evolutionarily conserved process in neoplastic transformation in human and chicken lymphomas of different etiologies [7]. Like human CD30^hi^ lymphomas, MD lymphomas are a heterogeneous mix of a minority of neoplastically transformed lymphocytes (CD30^hi^), surrounded by majority of non-transformed (CD30^lo^) lymphocytes [8,9]. Physiologically, CD30 signaling modulates cell survival and death; however, in CD30^hi^ lymphoma cells, it preferentially promotes cell survival [10,11]. CD30 overexpression (in both human and MD lymphomas) induces a T helper 2 (Th-2) or regulatory T cell (T-reg)-like cytokine microenvironment, which is antagonistic to cell mediated immunity, immune evasive [6,12,13], and promotes lymphomagenesis [8,14,15].

CD30 signaling activates the transcription factor Nuclear Factor-kappa B (NF-κB), which regulates genes associated with cell survival, proliferation, programmed cell death (PCD), stress and immunity [16]. Constitutive NF-κB activation, due to CD30 overexpression and ligand dependent/independent signaling, results in neoplastic transformation in human CD30^hi^ lymphomas [1,17]. The human oncogenic viruses Epstein-Barr virus (EBV/HHV-4) and Kaposi’s sarcoma-associated herpesvirus (KSHV/HHV-8) both subvert NF-κB activation via the CD30 signaling pathway when transforming cells—as MDV does in the chicken [18]. This suggests that the CD30 signaling pathway is fundamental, or at least highly beneficial to herpesvirus survival [18].

MD transformed lymphocytes have increased MDV oncogene “Meq” expression [19]. Meq is essential for MDV lymphomagenesis [20,21] and a positive correlation exists between Meq and CD30 expression [18]. Also, the chicken CD30 promoter has 15 known Meq binding sites, and Meq’s promoter has at least one NF-κB binding site [18]. We hypothesize that a feed-forward loop exists, with Meq induced CD30 overexpression, constitutive NF-κB activation with resulting increased Meq transcription—favoring neoplastic transformation.

Here we show, using MD lymphocytes isolated directly ex vivo that they are either neoplastically transformed and express high levels of CD30 (CD30^hi^) or are non-transformed and express low levels of CD30 (CD30^lo^) that: 1) neoplastic transformation is a continuum and the CD30^lo^ lymphocytes within the tumor microenvironment are pre-neoplastic; 2) as the lymphocytes become more neoplastically transformed they become more immune-evasive; 3) the MDV oncogene Meq, has a direct role in this process and 4) NF-κB has a central role in this neoplastic transformation. In vitro*,* we show that: 1) a feed forward loop exists in which Meq activates CD30 transcription resulting in CD30 protein overexpression, which induces NF-κB activation which activates Meq transcription (in addition to other genes); 2) Meq and NF-κB transcriptional effects on the Meq promoter can be additive and that NF-κB isoforms have different effects; 3) Meq transcriptionally activates or represses the CD30 promoter depending on whether it is derived from a MD-susceptible or -resistant genotype; 4) the Meq interactome consists of proteins involved in physiological processes central to lymphomagenesis.

## Results and discussion

Because the proteome directly affects phenotype, but the transcriptome merely influences the proteome and thus may only indirectly affect the phenotype [22-24], we based our systems biology model of neoplastic-transformation in MD on the differences between the transformed CD30^hi^, and the non-transformed CD30^lo^ MD lymphocytes proteomes. We isolated CD30^hi^ and CD30^lo^ lymphocytes directly ex vivo at >99% purity as described [12]. All comparisons and differential expressions are expressed as CD30^hi^ relative to CD30^lo^ lymphocytes (at p < 0.05). Of the 11,958 proteins we identified (Additional file 1, PRIDE Accession # 14847–14852) 1,588 proteins were significantly increased, and 808 proteins had significantly decreased expression in the CD30^hi^ lymphocytes.

### Functional modeling

**Figure 1:**
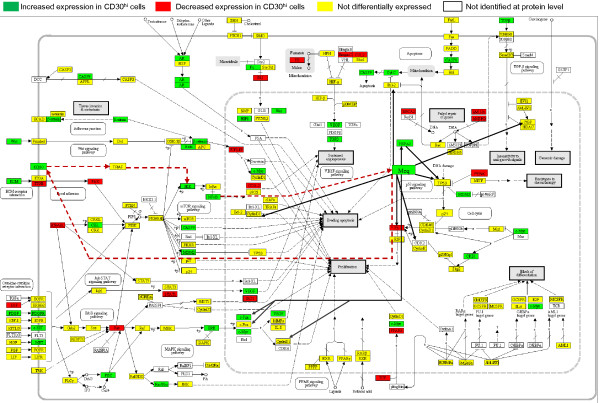
**Proteomics based network model showing differential expression of proteins in canonical cancer pathways and the intricate involvement of Meq, via hypothesized CD30-NF-κB-Meq pathway.** Canonical Network model of proteome data using differential proteomic expression (CD30^hi^ compared to CD30^lo^) mapped against cancer specific KEGG pathway “Pathways in cancer” and further modified by adding published Meq interaction and hypothesized Meq-CD30-NF-κB-Meq pathway. Light black solid lines indicate direct interaction, dotted lines indicate indirect interaction. Red dotted lines indicate our hypothesized CD30-NF-κB-Meq pathway. Literature based Meq interacting proteins are indicated by dark black lines originating from Meq.

To visualize the differences between the CD30^hi^ and CD30^lo^ lymphocytes proteomes (Figure 1) in terms of well-studied cancer pathways, the differential protein expression data (Additional file 1) was manually mapped to the cancer specific pathway “Pathways in cancer” from the Kyoto Encyclopedia of Genes and Genomes (KEGG) [25] (Figure 1). This specific KEGG pathway is a map of several different interacting signaling pathways and so provides a comprehensive overview of the molecular signatures of CD30^hi^ and CD30^lo^ lymphocyte proteomes. We further modified the KEGG pathway by adding the Meq oncoprotein, previously published Meq interacting proteins, and our hypothesized Meq-CD30-NF-κB feed forward loop. 

A mixed pattern emerged with protein levels increasing, decreasing and not changing. However, in several of the pathways described below, key regulatory proteins were differentially expressed: NF-κB, IKK, VEGF, MDM2, CD30, HSPA2, MYC, JUN, TGFβ, and Meq were increased; whereas, RB, PENK, and BRCA2 were decreased. This indicates that neoplastic transformation is being regulated by these key proteins. The MDV oncoprotein Meq interactions, and our hypothesized Meq-CD30-NF-κB feed-forward loop, suggest that Meq interacts with several key proteins involved in neoplastic transformation, immune evasion and cell survival.

Ingenuity Pathway Analysis ( http://www.ingenuity.com, IPA)-based functional grouping of the significantly expressed pathways (data not shown) confirmed our previous findings [26] that PCD was perturbed and integrin signaling was increased in CD30^hi^ cells. IPA analysis also indicated that PCD signaling, molecular mechanisms of cancer, NF-κB activation by viruses, p53 signaling, PPARα/RXRα activation, PTEN signaling, BRCA1 in DNA damage, VEGF signaling, Wnt/β-catenin signaling, lymphotoxin β receptor signaling (important in non-canonical NF-κB activation pathway induction), TGF-β signaling (insensitivity to antigrowth signals) and nitric oxide signaling (important in angiogenesis) were activated in both CD30^hi^ and CD30^lo^ cells. The physiological processes that the pathways affect, and the differences between the cell types, suggest that the CD30^lo^ lymphocytes are pre-neoplastic precursors of the CD30^hi^ lymphocytes.

To this point our modeling was on a global scale. Using the same data (Additional file 1), we next tested eight specific functional hypotheses (*a-h,* below) pertaining to essential steps of neoplastic transformation in the transition of CD30^lo^ to CD30^hi^ lymphocytes:

a) *Growth signals are perturbed*: Growth factors control cell division and their deregulation contributes to neoplasia. IGF1 increases cell cycle and prevents PCD [27] and it is transactivated by GH1 (via STAT5 [28]). Growth hormone GH1, which interacts with MDV’s SORF2 protein, is a suggested MD resistance gene [29]; however, both GH1 and SORF2 protein expression were the same in the CD30^lo^ and in CD30^hi^ cells. Our results suggest that the growth factor effects on MD resistance identified previously (32), may either occur at an earlier stage of MD, or are unrelated to lymphomagenesis. Growth factor receptors activate pathways for growth, proliferation, differentiation, survival, migration, angiogenesis and metabolism and, in contrast to the growth factors, the growth factor receptor proteins HGFR (MET) and PDGFR were increased. HGFR, which binds FAS and inhibits PCD, is also over-expressed in human CD30^hi^ lymphomas [30-34] as is PDGFR [35-37]. PDGFR over-expression can also make cells hyper-responsive to PDGF [36]. CD30^hi^ lymphocytes also had 4-fold more nuclear-located ERBB protein and over-expression and nuclear localization of ERBB-1 and −2 are common in tumors [38].

Growth factor receptors activate the MAPK, JAK-STAT, and, through PI3K/AKT, the MTOR signaling pathways. The MAPK pathway activates JUN, FOS and MYC, and the JAK-STAT pathway activates VEGF and both promote proliferation and angiogenesis. In the MAPK pathway, HRAS was decreased and JUN and MYC were increased. JUN mRNA was decreased and, as JUN transcription is autoregulated by JUN protein (45), and JUN heterodimerizes with Meq. We suggest that even though total JUN protein was increased in CD30^hi^ lymphocytes, it is not available for auto-transactivation, an alternative possibility is that as JUN protein is stabilized by post-translational interactions with Meq, the JUN mRNA may not actually reflect the total JUN protein levels [39].

Activated PI3K phosphorylates AKT, which in turn activates IKKA, MTOR (FRAP1) and MDM2 and inhibits FKHR, CASP9, BAD, p27 and p21 genes [40-42]. IKKA, MDM2, CASP9 increased, though FKHR, p27, p21, MTOR (FRAP1) did not. PTEN inhibits PI3K signaling in the absence of growth factors, and STK11 (LKB1) inhibits MTOR activity when ATP is low [40]. Consequently, cells lacking functional PTEN or STK11 exhibit deregulated, but constitutive, signaling to MTOR, resulting in cancer (46, 47). Though PTEN protein was not differentially expressed, STK11 protein (a tumor suppressor) decreased. From an antigrowth signal perspective, RB1 sequesters the E2F transcription factors transcriptionally-repressing genes essential for G1 to S phase cell cycle progression [42,43] and RB1 was decreased suggesting increased cell cycle progression in CD30^hi^ lymphocytes supporting our previous work [15].

b) *Cell cycle and PCD are dysregulated*: Cell cycle regulation and PCD are intimately linked. The proto-oncogenic WNT proteins were increased and WNT activation leads to CTNNB protein nuclear translocation. CTNNB also increased and was 80% nuclear. Canonically, CTNNB translocation results in TCF-mediated activation of the proto-oncogene MYC (pro-cell cycle/anti-PCD), anti-PCD protein SURVIVIN and the G1/S-specific cyclin-D1 (CCND1) [44,45]. BCL2 blocks apoptosis in many diverse cancers, and in-vitro work using a rodent fibroblast cell line, suggests that MDV Meq increases BCL2 mRNA [46], and proposed that this is important in MD lymphomagenesis. In our work from MD lymphocytes in vivo, BCL2 protein was unchanged suggesting that any BCL2 functional-deregulation may occur prior to the CD30^lo^ to CD30^hi^ transition in the lymphoma environment. HSP70 inhibits both the intrinsic and the extrinsic PCD mechanisms and is frequently increased in malignant tumors [47-50], Meq also co-localizes with HSP70 in the nucleus [49] where HSP70 mediates Meq’s interaction with TP53 and CDK2 [49]. In agreement, we found HSP70 protein was increased and was 100% nuclear. Decreased PENK increases anti-PCD-gene transcription [51] and PENK protein was decreased by half, and its nuclear distribution decreased by 70%, suggest decreased PCD possibly mediated by Meq (see below).

c) *Telomeres are dysregulated:* Shortened telomeres promote PCD and the telomerase complex maintains telomere length in cancer [52]. The telomerase complex has two core components: telomerase RNA (TR; the template for telomere repeat synthesis) and the enzyme TERT. CD30^hi^ lymphocytes have 20% more nuclear TERT. Furthermore, POT1, a protein also required for telomerase maintenance [53,54], was also increased in CD30^hi^ cells.

d) A*ngiogenesis is increased****:*** Tumor cells can induce neo-angiogenesis or vasculogenesis [55], and pro-angiogenic VEGF was increased and anti-angiogenic MMP9 remained unchanged, suggesting endothelial cell proliferation and angiogenesis.

e) *Metastasis is promoted:* Metastasis a primary cause of cancer mortality and part of MD pathogenesis. Ezrin (EZR) is essential for metastasis [56] and is consistently increased in metastatic cancers [57]. EZR complexes with NF2, links membrane proteins and the actin cytoskeleton, and regulates cell survival, adhesion and migration [58]; it also complexes with CD44 [59] and MET [60,61] to promote metastasis. EZR, NF2, CD44 and MET were all increased suggesting that metastasis is more a function of CD30^hi^, than CD30^lo^, lymphocytes and this is consistent with human CD30^hi^ lymphomas.

f) *Immune evasion mechanisms are increased:* MAN1A2, (which induces T-reg cell migration and prevents T cell priming [62]), was increased and this supports our previous contention that as neoplastic transformation proceeds, a T-reg-like phenotype is induced [3,6,12]. IRG1 protein and mRNA (p < 0.06) were decreased in the CD30^hi^ cells. Expression of IRG1 mRNA is induced by pro-inflammatory cytokines and lipopolysaccharide after bacterial infection of macrophages/monocytes [63]. There is very limited published literature about IRG1’s functions in lymphomas, however, recent studies in MD literature have suggested that IRG1 may be associated with apoptosis and is potentially pro-apoptotic [64]. By searching the EBI *Gene Expression Atlas* ( http://www.ebi.ac.uk/gxa/) we found that IRG1 mRNA is decreased in some human and mouse lymphoid neoplasia datasets also—as is its regulator leukemia inhibitory factor (LIF; which was decreased at the protein level in our data). We speculate that both LIF and IRG1 are worthy of investigation in future for a role in neoplastic transformation and anti-apoptosis in MDV pathogenesis [64]. The data that we found in the EBI *Gene Expression Atlas* shows that such a mechanism may exist in human disease also, but this data has not yet been recognized, nor the hypothesis tested, by human medical research.

g) *Epigenetic regulators are activated:* DNA methyl transferases (DNMT1, DNMT3A and DNMT3B), histone acetyltransferase (HAT) and histone deacetylases (HDAC)] are implicated in human and MD lymphomas [65] and HDAC-8 and −10 mRNAs, and DNMT3B and HDAC9 proteins, were increased.

h) *MDV proteins other than Meq are involved and have altered expression:* The MDV DNA replication genes thymidine kinase and deoxyuridine triphosphatase (73–75) decreased, in agreement with MDV being latent; but, in addition to Meq being increased, so were the envelop glycoproteins D, I, K, the major capsid protein and nuclear egress lamina protein – all are structural proteins important for MDV horizontal transmission. This supports our previous work that CD30^hi^ lymphocytes have the highest load in lymphomas [66] and suggests lateral MDV cell-cell transmission within the lymphoma. We speculate, that MDV, like EBV [67] has more than one “latency program” and that the immuno-suppressive lymphoma environment “permits” MDV to produce more proteins than it would in other environments. We also suggest, based on our data above, that, as in EBV [68], epigenetic regulation plays a role in latency programs.

### Biological processes associated with neoplastic transformation and immune-evasion

**Figure 2:**
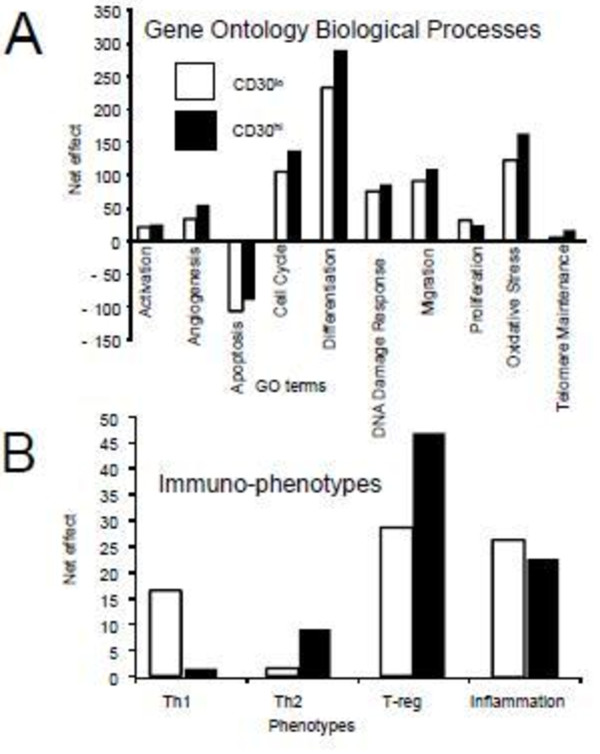
**Proteomics based Gene ontology (GO) based quantitative modeling of cellular immunophenotypes of CD30**^**lo**^**and CD30**^**hi**^**cells.** Quantitative modeling of the CD3^lo^ and CD30^hi^ lymphocyte proteome by Gene Ontology Biological Processes (**A**) and immunophenotype using *GO-modeler*[12] as described in methods [3].

At a higher level, the *Gene Ontology* (GO) allows explicit modeling not limited by canonical pathways [69]. We compared CD30^hi^ and CD30^lo^ lymphocyte proteomes, using quantitative GO biological process (BP) modeling (Figure 2A), for the biological processes inherent in neoplasia as described [3]. Although both the CD30^hi^ and CD30^lo^ lymphocytes have pro-neoplastic phenotypes (i.e. pro-cell activation, pro-angiogenic, anti-PCD, pro-cell cycle, pro-differentiation, pro-DNA damage response, pro-migration, pro-proliferation, pro-oxidative stress and pro-telomerase maintenance) the CD30^hi^ cell proteome is more pro-neoplastic than the CD30^lo^. 

Next, we compared the CD30^hi^ and CD30^lo^ lymphoma cell immune-phenotypes [6,12] (Figure 2B). We have identified the MD lymphoma microenvironment as predominantly T-reg-like [6] but did not differentiate which lymphocytes were contributing to the phenotype. Here we show that the CD30^hi^ and CD30^lo^ cell proteomes have similar T-reg-like phenotypes and the CD30^hi^ lymphocytes are more Th-2-biased, but less Th-1 and pro-inflammatory-biased, than the CD30^lo^ lymphocytes. This is consistent with a model of increased CD30 expression and signaling promoting immune-evasion [9,70].

### Transcriptional regulation

**Figure 3:**
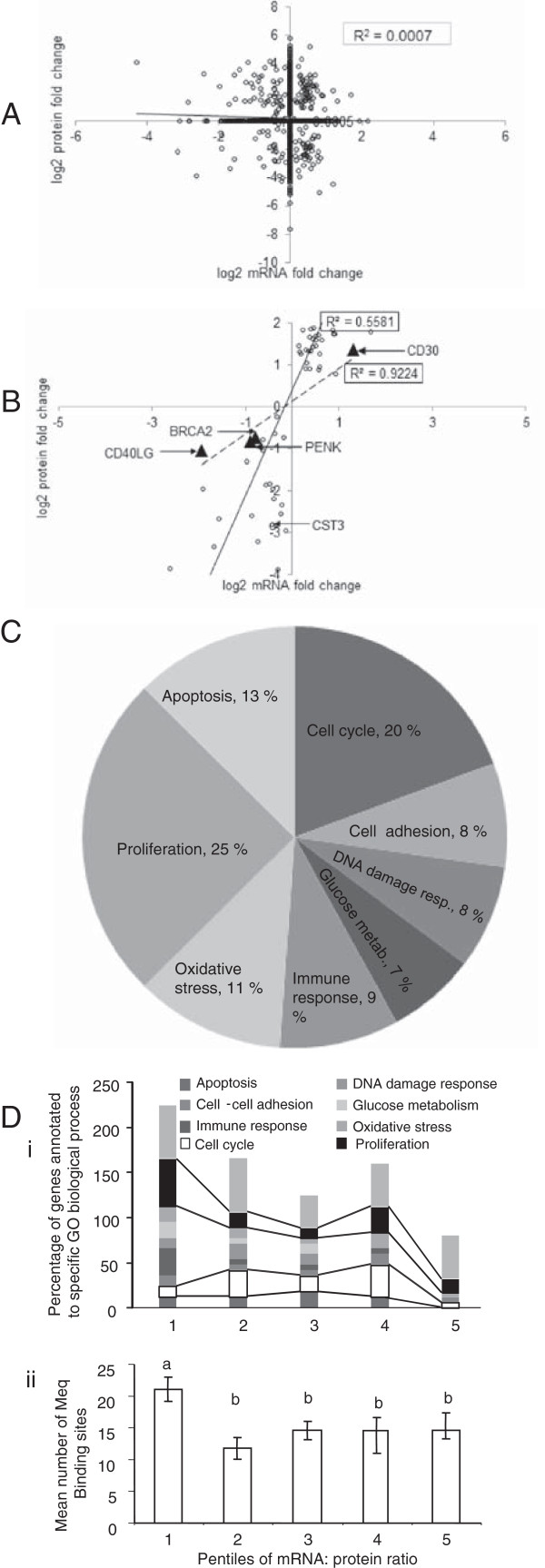
**mRNA and protein correlation and GO based modeling of 88 concordantly expressed host gene products.** Overall correlation (*R*^*2*^ = 0.0007) of protein and mRNA fold changes in CD30^hi^, compared to CD30^lo^ lymphocytes ((**A**); n = 4592). Overall correlation for gene products differentially expressed in the same direction at both mRNA and protein levels showing higher correlation (n = 88, *R*^*2*^ = 0.5581). The triangles indicate genes known to be involved in CD30^hi^ lymphomas, and have mRNA: protein correlation close to 1 (*R*^*2*^ = 0.92). CST3 is also involved in in CD30^hi^ lymphomas but has very low mRNA: protein correlation. (**B**). Gene Ontology (GO) Biological Processes (BP) associated with the concordantly-expressed gene products indicates involvement in cellular processes perturbed in neoplastic transformation (**C**). Concordant gene products were ranked based mRNA: protein correlation and then grouped into pentiles and distribution of GO BP by pentile were compared. Cell cycle and proliferation are disproportionately represented in pentile 1 (D-i). Pentile 1 genes have more putative Meq binding sites in their promoters than genes in the other pentiles (mean ± sem) (D-ii).

To identify potential direct transcriptional proteome regulation, we used the 44 K Agilent chicken microarray [71,72] to quantify mRNA and micro (mi)RNA (Additional file 2) isolated from the same CD30^hi^ and CD30^lo^ lymphocytes which were used for proteomics and compared transcriptional fold changes with protein fold changes (Figure 3A; Additional file 3). Overall there was poor fold change correlation between mRNA and protein for 4592 host gene products (*R*^*2*^ = 0.0007). Next, to identify the key regulatory proteins responsible for neoplastic transformation, all the gene products which were differentially expressed in the same direction at both mRNA and protein levels were selected for further analysis. There are 88 gene products whose mRNA and protein fold changes were both significant and directionally consistent with each other (i.e. concordant) and these have an overall positive correlation (Figure 3B, *R*^*2*^*=* 0.5581). Of these, on cross referencing with the published literature, revealed that BRCA2, CD30, CD40L, CST3 and PENK are known to be involved in human CD30^hi^ lymphomas [17,51,73-79] and, except for CD30, all had decreased expression in CD30^hi^ cells. BRCA2 is involved in error-free DNA-damage repair and decreased BRCA2 expression results in erroneous joining of DNA breaks [73]; CD30 is over-expressed in all human HL and some NHL [74,80]; CD40L prevents caspase-dependent and -independent PCD in HL cell lines [75]; CST3 is secreted by neoplastically transformed cells [76], inhibits neovascularization [81] and, via its inhibitory effect on cathepsin B and S, inhibits tumor invasion and metastasis [82] and is a biomarker in humans for NHL relapse [77]. CST3’s mRNA and protein decrease in MD CD30^hi^ lymphocytes is consistent with human and murine lymphomas [77-79] and decreased CST3, enhances angiogenesis, tumor burden, tumor cell proliferation and tumor invasion [83] and also leads to increased expression of pro-neoplastic growth factor like IGF1 and FGF1 in mice [84]. In cells over-expressing NF-κB, and in coordination with TP53, PENK induces PCD [51], and so its decreased expression favors neoplasia. 

Specific GO-based BP modeling of these 88 concordantly-expressed genes shows that they are involved in BPs known to be perturbed in, and central to, neoplastic transformation (Figure 3C): 25% are involved in proliferation, 20% in cell cycle and ~ 10% in regulating PCD, cell-cell adhesion (essential for metastasis), innate and adaptive immunity, oxidative stress, DNA damage response and glucose metabolism.

We next ranked the genes based on their mRNA: protein expression correlation, and then grouped them into pentiles and compared the distribution of BP by pentile (Figure 3D-i). Across the five pentiles gene expression regulation was the most dominant BP; the next two biggest BP groups, consistent across the five pentiles, were proliferation and cell cycle. Both proliferation and cell cycle are central to lymphoblastoid cell physiology and neoplastic transformation. The proliferation: cell cycle and proliferation: PCD ratios were both 4.5 in pentile 1. In contrast the mean ratios for the other four pentiles were 1.4 (range 0.6 to 3). The high correlation between mRNA and protein expression, coupled with predominance of genes involved in cell proliferation in pentile 1 (compared to other four pentiles), suggested that pentile 1 genes may be transcriptionally regulated via Meq and this would favor neoplastic transformation.

We next identified the numbers of putative canonical MDV Meq binding sites (searched for the core AP-1, MERE I and MERE II motifs as described in [85]) in each of the 88 concordantly-expressed genes’ promoters (2.5 Kb 5’ of the ATG) as described [86]. Genes in pentile 1 (with mRNA: protein expression ratios closest to 1:1) have more Meq binding sites in their promoters than those in the other pentiles, which do not differ from each other (Figure 3D-ii, Additional file 4). Of the five concordant genes previously implicated in lymphomagenesis in other species, BRCA2, CD30, CD40LG, and PENK are in pentile 1 with a group mRNA:protein expression correlation (*R*^*2*^) of 0.92, suggesting direct transcriptional regulation by Meq. In contrast, CST3 is in pentile 4 with a large decrease in protein but small decrease in mRNA. It is possible that CST3 is regulated at the level of miRNA; an alternative possibility is that CST3 is a secreted protein so a small decrease in mRNA could result in a large decrease in cellular protein and, consistent with our observation, most CST3 was located in the predominantly soluble differential detergent fraction 1. Notably, IRG1 was in pentile 1, and has the most Meq binding sites of all the concordant genes (51), all of which are MERE II binding sites, suggesting Meq induced transcriptional repression, and a central role in MD neoplasia. Overall, the data suggests that the genes in pentile 1 are critical for neoplastic transformation.

miRNAs are non-coding post transcriptional repressors potentially important in neoplasia and we identified 152 expressed chicken miRNAs (Additional file 2). Of these, nine (gga-mir-1b, gga-mir-7, gga-mir-7b, gga-mir-10b, gga-mir-31, gga-mir-130b, gga-mir-204, gga-mir-215, gga-mir-489) are increased, and five (gga-mir-223, gga-mir-124b, gga-mir-140, gga-mir-183, gga-mir-222a) are decreased in CD30^hi^ cells. In MDV infected CEFs, gga-mir-29b,-196,-133a,-10b,-30d were increased, and gga-mir-let-7a, 7b, 7f and gga-mir-1a, mir-130a were decreased [87]; of these only gga-mir-10b was increased in our data. This suggests that the in vivo lymphoma environment where MDV is “latent” is functionally quite different from a lytic fibroblast culture. In vitro, gga-mir-221 and gga-mir-222 inhibit expression of CDK inhibitor protein p27/KIP1, but p27/KIP1 protein was increased in the MDV transformed lymphoblastoid cell line MSB-1 [88]. In our results gga-mir-221 was not differentially expressed and gga-mir-222a was decreased: and this is consistent with our data that p27/KIP1 protein is not differentially expressed. gga-mir-26a inhibited IL-2 mRNA and was decreased in seven MD transformed cell lines [89,90], but again in our dataset, neither gga-mir-26a nor IL-2 were differentially expressed and neither was IL-2 protein.

We used the *miRDB*[91,92] to identify novel miRNA targets (Additional file 2), and we found that the 9 different miRNAs that increased in CD30^hi^ lymphocytes target several genes associated with neoplastic processes (Additional file 2): gga-mir-204 targets FAS apoptosis inhibitory molecule 2, RAB22A (a RAS oncogene family member) and HDAC 9; gga-mir-489 targets FAS associated factor 1 (FAF1) and gga-mir-7 targets RAS related viral oncogene homolog 2. Except FAF1 (which was unchanged) none of these proteins were identified and so we cannot confirm the upregulated miRNA’s potential effects on neoplasia in CD30^hi^ cells. Notably however, gga-mir-183 which targets EZR mRNA (which did not change), was decreased and EZR (important in metastasis) protein increased; i.e. we suggest that one reason for the increase in EZR protein is decreased gga-mir-183 translation-inhibition.

### CD30^hi^ lymphocytes have increased levels of activated NF-κB

Constitutive NF-κB activation is a proposed mechanism by which overexpressed CD30 induces neoplastic transformation in human HL and NHL and in MD [18]. Our global proteomics modeling data (Figure 1), Ingenuity Pathway analysis, and mRNA-protein correlation data (Figure 3) further suggested a direct role of Meq and NF-κB in MD transformation. CD30 activates NF-κB via both canonical and non-canonical pathways and both ligand-dependently and -independently [11]. In the canonical pathway, IκB inhibitors, IκBα, IκBβ, and IκBϵ (not identified in chicken) are phosphorylated by IκB kinases (IKK) and ubiquitinated by ubiquitin ligase (BTRCP). Proteasomal degradation of IκB inhibitory proteins releases NF-κB dimers, which translocate to the nucleus and transactivate target genes [93]. In the non-canonical pathway, p100 (NF-κB2 precursor protein) acts as IκB inhibitory molecule and an IKKα homodimer acts as the main activator: IKKα phosphorylates p100, resulting in proteasomal degradation of inhibitory C-terminal domain (IκBδ), which generates the p52 subunit (functional NF-κB2 protein) and dimerizes with RelA or RelB to form functional NF-κB dimers [94].

**Figure 4:**
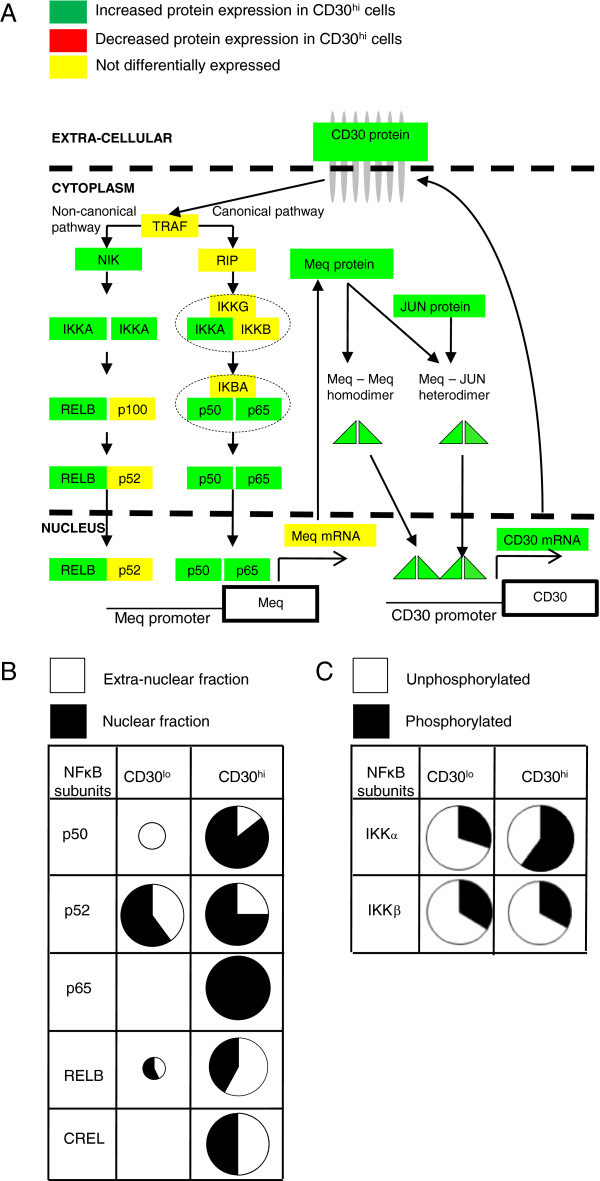
**Schematic diagram of the hypothesized Meq-CD30- NF-κB feed forward loop, subcellular localization and phosphorylation status of different isoforms of NF-κB.** Schematic diagram of hypothesized Meq-CD30-NF-κB feed forward loop and showing differential expression of NF-κB isoforms (**A**). Comparison of the amount and sub cellular localization of NF-κB isoforms: circle size proportionately portrays relative protein amount; equal size indicates no differential expression at P < 0.05 (**B**). In CD30^hi^ lymphocytes most IKKα is phosphorylated at the canonical residues that regulate proteasome-mediated degradation and destabilization, whereas in CD30^lo^ lymphocytes most IKKα and IKKβ is unphosphorylated at these same residues (**C**).

We found that NF-κB p50 (functional NF-κB1), p65 (RelA) and RelB and IKKα proteins all increased in CD30^hi^ lymphocytes (Figure 4A) and most p50 and all p65 protein (which form the most common and abundant classical dimers) were nuclear (Figure 4B, Additional file 1). NF-κB signaling is controlled by negative feedback via IκBα and A20/TNIP2 (tumor necrosis factor alpha-induced protein 3 [TNFAIP3] in chicken) transcriptional induction [95] and we found TNFAIP3 mRNA and protein unchanged but IκBα mRNA decreased, suggesting that this negative feedback mechanism is suppressed. The TNFAIP3 and IκBα promoters have 18 (all MERE II) and 9 (3 MERE I and 6 MERE II) predicted Meq-binding sites, respectively, which suggest that MDV has evolved to maintain NF-κB activation. Not only do CD30^hi^ lymphocytes have more of all NF-κB isoforms but more are nuclear (Figure 4B, Additional file 1), again suggesting NF-κB activation. Furthermore in CD30^hi^ lymphocytes, most IKKα is phosphorylated at the canonical residues that regulate proteasome-mediated degradation [96,97] and destabilization [98-100], whereas the opposite occurred for IKKα in CD30^lo^ lymphocytes (Figure 4C, Additional file 1). 

### NF-κB transactivates Meq transcription in vitro

**Figure 5:**
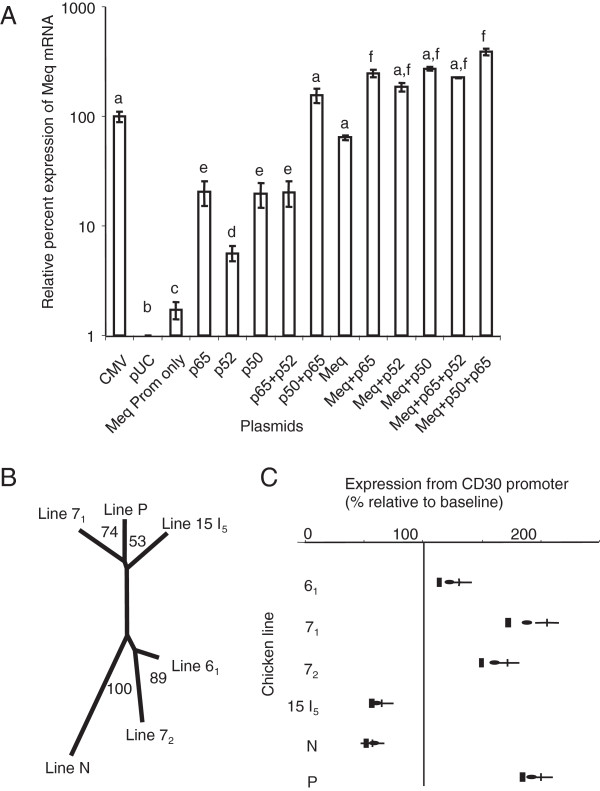
**QPCR based results of various transcription reporter assays and phylogenetic tree constructed from CD30 promoter sequences of MD resistant and susceptible genotypes.** QPCR results from in vitro transcription assay comparing the efficacy of NF-κB subunits and Meq combinations on transcription from the MDV RB-1B Meq promoter (mean ± sem); a-f indicate statistical differences (**A**). Unrooted phylogenetic tree constructed from CD30 promoter sequences for MD-resistant (6_1_, N) and MD-susceptible genotypes (7_,_ 7_2_, 15I_5_ and P) (**B**). Quantitative effects of Meq on transcription from the MD resistant and susceptible genotypes’ CD30 promoter (mean, 95% CI) (**C**).

Because we proposed a feed-forward loop model of increasing Meq and CD30 expression [18] and our global analysis suggests that NF-κB is central in MD lymphomagenesis, we tested NF-κB isoforms’ transactivation potential on the Meq promoter using in vitro transcription reporter assays (1 Kb 5’ of the Meq ATG; MDV strain RB-1B). We cloned genes RELA [p65], NFKB1 [p105/p50] and NFKB2 [p100/p52] and MEQ (RB-1B) into expression plasmids. SOgE cells [101] were transfected with the reporter plasmid alone or in combination with plasmids expressing different NF-κB isoforms and/or Meq, and transcription was quantified by QPCR. The three NF-κB isoforms differentially transactivated the Meq promoter (Figure 5A): p52 was less than p50 and RELA (p65) alone, which produced similar transcription and were less than p50 and RELA (p65) together (in vivo p50 and p65 form the classical dimer). Meq alone transactivated the Meq promoter to similar levels as the positive control cytomegalovirus promoter and, when used together with different NF-κB isoforms, except in the p50-p65 dimer, it further increased transcription. This finding suggests that neoplastic transformation in MD depends fundamentally on CD30 signaling, and may explain why MD neoplastically-transformed cell survival (like that of many human herpesvirus induced lymphomas) critically depends on the lymphoma environment [6,12,15], as well as why MDV co-opted the CD30 signaling pathway. 

### Meq-dependent differential CD30 promoter transcription

It would be reasonable that differences in the CD30 promoter could confer differences in Meq-induced activation or repression of the CD30 gene and is of interest to us because of chicken genotype differences to MD lymphomagenesis after MDV infection. To measure Meq-induced CD30 transcription on different CD30 promoters, we first cloned and sequenced CD30 promoters (2431–2438 bp 5’ to the ATG) from two MD-resistant (lines 6_1_ and N) and four MD-susceptible (line 7_1_, 7_2_, 15I_5_, and P) genotypes of chickens and sequenced these. An unrooted phylogenetic tree (Figure 5B) of these sequences matched the chicken line breeding history [102]. Lines 6, 7 and 15 are part of 15 lines developed to study the genetics of avian neoplasia [102]. Line 6 and 7 share common ancestors and this is emulated in their phylogenetic closeness in our data. Line 15 is also genetically related to lines 6 and 7 and some line 15 birds were isolated and interbred to produce the 15I sublines. Further sublines (e.g. 6_1_, L7_1_, L7_2_ and 15I_5_) were produced by further inbreeding. Notably, line 7_1_ was accidentally crossed with 15I_5_[102,103], and we independently identified this event in our phylogenetic tree, which places Line 7_1_ closer to Line 15I_5_ than Line 7_2_. Lines N and P are non-inbred lines developed independently to study MHC class I-based resistance and susceptibility to MD [103,104].

After cloning into an expression plasmid, each CD30 promoter was used in in vitro transcription assays using a Meq-expressing plasmid. Meq altered transcription from all CD30 promoters’ alleles (Figure 5C) — increasing expression in MD-susceptible lines 7_1_, 7_2_ and P, but decreasing in the MHC MD-resistant line N and the very late lymphoma forming [9] line 15I_5_. MD resistant line 6_1_ had a small increase in transcription. The trend is that CD30 promoter transcription is associated with MD lymphoma resistance and susceptibility and that Meq has host genotype-dependent transcriptional-activation or repression from the CD30 promoter. However, although there are 56 single nucleotide polymorphisms (SNPs) between the lines’ promoter sequences (SNPs; NCBI accession numbers: EU000367-EU000372), none occur in the predicted canonical Meq binding sites [85] and sequences other than these previously-described Meq binding sites must be functional. We identified one SNP at position 1754 bp in 15I_5_ and 1755 bp in line N 5’ of the ATG as a candidate; transcription factor binding prediction [105] identifies the corresponding region in all lines as an AP-1 binding site and we suggest that this SNP could be responsible for differential function.

### Meq interacts directly with proteins central to lymphomagenesis

**Figure 6:**
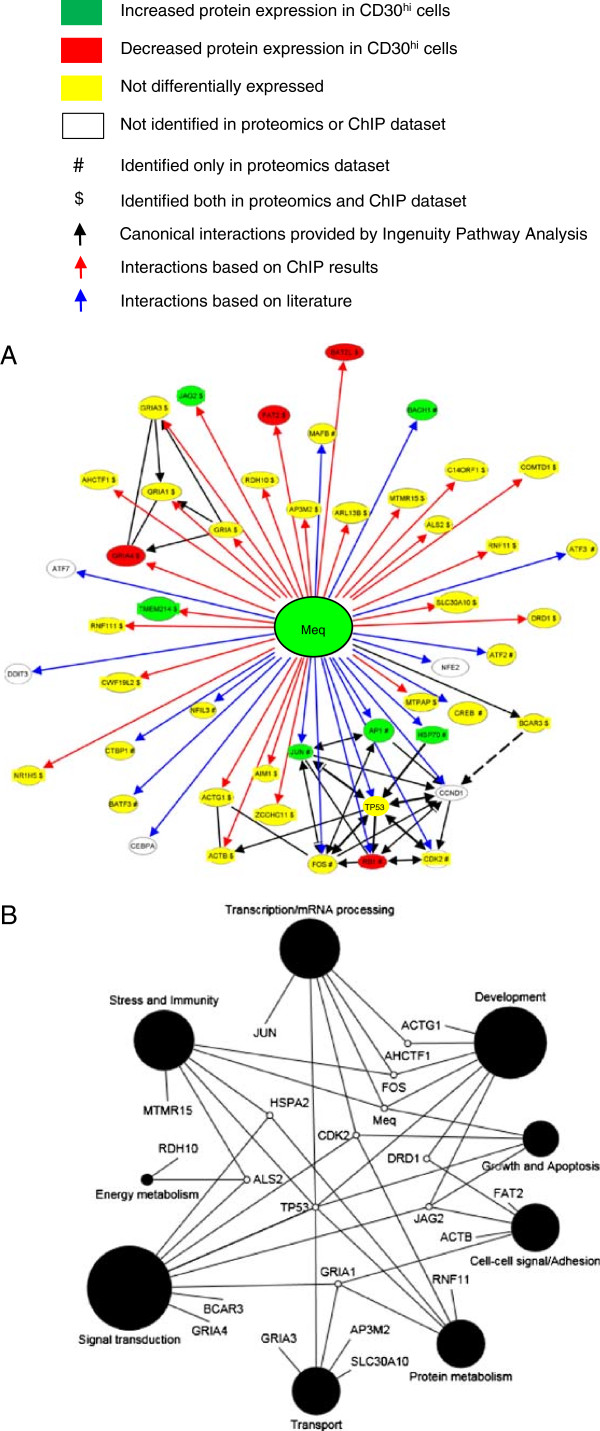
**Meq interactome showing previously published and novel interactions identified by ChIP experiments and GO based modeling of Meq interacting proteins.** Meq interactome based on combined data from proteins identified by chromatin immunoprecipitation (ChIP) experiments (see Methods) and published literature. Differential expression is shown for proteins identified by ChIP. (**A**). Meq interactome converted to a GO functional network showing the physiological processes that Meq directly affects are consistent with neoplasia. Circle size represents effect size based on numbers of proteins associated with that GO annotation (**B**).

Meq’s functions are modulated by its interacting partners. Here we wanted to identify which proteins were involved with Meq in the context of DNA binding and so we used chromatin immunoprecipitaion (ChIP) using anti-Meq antibodies (rather than traditional immuneprecipitation), followed by 2D LC-MSMS. We used MSB-1 MDCC cells as a model for tumor cells. We identified 31 proteins (PRIDE Accession # 14847–14852, Additional file 1). We used these 31 proteins and included previously identified interacting proteins (RB1, TP53, CTBP, HSP70, JUN, FOS, CDK2, CCND1), to produce theoretical Meq interactome model. From these, and using binding proteins from literature, we produced a Meq interactome model (Figure 6A). Using GO BP annotations for all the proteins that we modeled in the network, we next generated a GO BP-based functional interaction network (Figure 6B). This model suggests how Meq could interact with proteins associated with BPs critical to tumor formation such as cell growth, development, apoptosis, stress, immunity, transcription, cell adhesion, energy metabolism, protein metabolism and transport.

## Discussion

Evidence supporting a direct mechanistic connection between inflammation and cancer has been mounting over the last decade [106]. The very early pre-lymphoma MD lesion microenvironments are highly inflammatory [6]. NF-κB is one of the central inflammatory mediators that is often, and diversely, associated with neoplastic transformation [107] and is a key component of the transformation pathways employed by some herpesviruses. The KSHV latency-associated proteins vGPCR and vFLIP, maintain a sustained level of activated NF-κB by interacting with IKK protein complex and micro RNA clusters (miR-K1) which inhibit IκBα protein expression, thus inhibiting the lytic cycle, inducing the latency and transformation [108-111]. In EBV positive HL the transformed cells overexpress CD30, CD40, RANK receptors and their cognate ligands are expressed on surrounding stromal cells. Ligand and receptor interaction (or sometimes ligand independent signaling) activates downstream signaling and activation of NF-κB occurs [112]. EBV encoded LMP-1 protein mimics the activated CD40 receptors and results in spontaneous NF-κB activation [67]. Our “omic” and reductionist experiments in this work suggest that MDV has also evolved to directly perturb the NF-κB signaling pathways while in viral latency.

In vitro MDV Meq induced CD30 expression and persistently activated NF-κB and ex vivo-derived CD30^hi^ lymphocytes have increased and activated NF-κB protein. Not only does Meq enhance its own transcription (as previously described [20]) but it also augments NF-κB transcription. We also suggest that IκBα-mediated negative feedback, which controls NF-κB activation, is hypoactive in CD30^hi^ cells. This is consistent with evidence that proinflammatory cytokines induce NF-κB inducing kinase (NIK), which preferentially phosphorylates IKKα over IKKβ to activate NF-κB [113] and, while recent evidence suggests that IKKβ is primarily activated in response to pro inflammatory cytokines and microbial products, IKKα regulates the alternative pathway of NF-κB activation in lymphoid malignancies [114]. IKKα is also preferentially activated by the members of TNF receptor family [115]. Inducing persistent NF-κB signaling through specific oncoproteins has been demonstrated for human oncogenic viruses, including EBV, human T cell leukemia virus type 1, and KSHV [116]. Notably, EBV LMP-1 effects NF-κB activation through the NF-κB essential modifier protein which, with IKKα and IKKβ protein, comprises the IκB kinase (IKK) complex [117] and we speculate that MDV (possibly via Meq) has evolved to similarly target the IKK complex.

Regardless, our data supports our hypothesized model that Meq initiates a self-reinforcing CD30 signaling cycle resulting in constitutive and aberrant NF-κB activation and subsequent neoplastic transformation. Herpesviruses co-evolve with their hosts and and the last common ancestor between EBV and MDV was at least ~300 M years ago; MDV, EBV and KSHV have separately evolved in different target cells the same fundamental result by targeting the NF-κB pathway. Furthermore both MDV Meq and EBV LMP-1 [118] are expressed as proteins during viral latency and their hosts mount specific (but weak) cytotoxic T cell responses against them [18,119,120]. This large evolutionary distance, combined with the risk incurred by inducing an immune response, suggests that perturbing NF-κB confers a strong evolutionary advantage and is further evidence consistent with NF-κB essentiality to neoplasia in general.

Meq is essential for MD lymphomagenesis [21,121] and promotes neoplastic transformation, anchorage-independent growth, cell-cycle progression, and anti-apoptotic activity [46,122-124]. Our in vitro experiments support Meq’s previously demonstrated transcriptional regulation of CD30 [18], and, also show that the transcriptional profile generally follows genetic resistance and susceptibility to MD. A similar phenomenon has been observed in the CD30 over-expressing human cutaneous lymphoproliferative disease lymphomatoid papulosis [4,125]: allelic differences in the CD30 transcription are due to polymorphisms in the human CD30 microsatellite repressor element (located −1.2 kb and −336 bp of the CD30 promoter) and are associated with disease progression to lymphoma [125].

Non-transformed cells are common in lymphomas of all species, and often they form the majority cells in lymphomas. Our work suggests that many of these non-transformed cells are likely not immune responding cells in MD [15], but are pre-neoplastic and actively transforming. Regardless, an immunosuppressive tumor microenvironment is critical in lymphomagenesis [6,8,12,14,15,126,127]. In EBV-positive HL, the lymphoma microenvironment is T-reg cell rich and the transformed cells secrete immunosuppressive cytokines and chemokines like IL10, CCL5, CCL20, and CXCL10. These cytokines and chemokines (may not always match classical T-reg cytokine profiles [128-130]), attract non-transformed cells [131,132] to the site of lymphomagenesis. Similarly, in MD, a recent study has shown interactions between vIL-8 (a chemotactic factor) and peripheral CD4 + CD25+ T cells (closely resemble T–reg phenotype) [12,133], and suggested that vIL-8 may enhance the recruitment of T-reg cells to the MDV lymphoma microenvironment, which would further induce immunosuppression and enhance lymphomagenesis, supporting our previous observations [6,12]. Here, we have expanded on our previous work [6] and show that both components of lymphoma microenvironment, the CD30^hi^ and CD30^lo^ cells have an overall T-reg-like phenotype and suggest that CD30^lo^ lymphocytes are direct antecedents of CD30^hi^ lymphocytes.

Overall, and in the context of understanding mechanistic details of CD30^hi^ lymphomagenesis, our results provide direct ex vivo-derived support in a natural animal model for the in vitro results in other species, which propose that ligand-independent and -dependent CD30 signaling-induced constitutive activation of NF-κB is a mechanism of neoplastic transformation in Hodgkin’s disease antigen over-expressing lymphomas [17].

## Conclusions

Here we identify the neoplastic and non-neoplastic component of lymphoma microenvironment using transcriptomics and proteomics followed by Systems Biology modeling to generates specific hypotheses and then tests these using reductionist methods. This work provides evidence that MD neoplastic transformation is a continuum and the CD30^lo^ lymphoma cells are in various stages of neoplastic transformation towards CD30^hi^ phenotype. We hypothesized that MDV uses its Meq oncogene to activate CD30 transcription to achieve constituent NF-κB signaling resulting in cellular instability and a neoplastic phenotype. Our results show that Meq, CD30 and NF-κB proteins are overexpressed in CD30^hi^ cells and that the majority of NF-κB is intranuclear suggesting an activated state. Using transcription reporter assays, we further show that NF-κB isoforms differentially activate Meq transcription, and Meq and NF-κB isoforms have additive effects. We also show that Meq transcriptionally activates or represses the CD30 promoter depending upon the host genotype from which the promoter is derived. Using ChIP and mass spectrometry we propose a new Meq interactome composed of proteins which are involved in various biological processes inherent in neoplasia. Overall, this study provides crucial insights into various molecular mechanisms of neoplastic transformation active within a heterogeneous lymphoma microenvironment in a natural animal model with functional immune system.

## Methods

### RNA isolation and microarray experiments

Lymphomas were isolated from white leghorn chickens infected with MDV GA/22 strain as described [9]. The CD30^hi^ and CD30^lo^ cells were separated using monoclonal antibody (mAb) AV37 using magnetic activated cell sorting (Militenyi Biotech) and the purity of sort was analyzed by flow cytometry as described [66]. RNA was isolated from 4 replicates of 10^6^ CD30^hi^ and CD30^lo^ lymphocytes using the TRI Reagent® (Molecular Research Center). The quality of purified RNA was analyzed using the Agilent 2100 Bioanalyzer (Agilent Technologies) and RNA was quantified using the GeneSpec I spectrophotometer [12,66]. The microarray design and methods have been described in [71,72]. Briefly, a 44 K Agilent chicken microarray with dual color balanced design was used [71]. The genes on the array included whole chicken genome, 150 chicken microRNAs, [72,134,135], all known MDV and two avian influenza virus (H5N2 and H5N3) transcripts [72]. 500 ng of total RNA was reverse transcribed into cDNA with a T7 sequence inserted in cDNA to drive the synthesis of complementary RNA (cRNA). The fluorescent labeled cRNA were purified, hybridized, washed and then scanned by Genepix 4100A scanner (Molecular Devices) with the tolerance of saturation setting of 0.005% [72]. The normalized data was analyzed using SAS 9.1.3 program (SAS Institute). An approximate F test on least-square means was used to identify the differentially expressed genes (p < 0.05) [72]. Data has been deposited in GEO database, accession numbers (Samples: GSM689844, GSM689863, GSM689864, GSM689865, Series: GSE28034).

### Protein isolation and protein analysis by 2 dimensional liquid chromatography electro-spray ionization tandem mass spectrometry (2D LC ESI MS/MS)

Proteins were isolated from three replicates from (from different birds) 10^7^ CD30^hi^ and CD30^lo^ cells using differential detergent fractionation (DDF), trypsin-digested and analyzed by 2D LC ESI MS/MS using a LCQ Deca XP Plus (Thermo Fisher Scientific) as described [136]. The experimental mass spectra and tandem mass spectra were searched (Bioworks, 3.2, Thermo Fisher Scientific), against an *in silico* trypsin digested non redundant protein database which included all annotated chicken and MDV proteins, with search criteria as described [3]. Peptide identification used decoy database searching and only peptides identified with p < 0.05 were used for further analysis; the differentially expressed proteins were then identified at p < 0.05 as described [137]. Data has been deposited in PRIDE database accession numbers 14847–14852. We searched the mass spectra for evidence phosphorylation of the conserved canonical residues regulating proteasome-mediated degradation [96,97] and destabilization [98-100] of inhibitor of nuclear factor kappa-B kinase (IKK)-α and IKK-β exactly as for non-modified peptides except that we searched explicitly for an additional 80 Da added to unphosphorylated amino acids [138,139] and calculated probabilities for phosphopeptides using decoy database searching, the degree of phosphorylation, as described [137,139].

### Co-immmunoprecipitation of Meq interacting proteins

Meq interacting proteins were identified by chromatin immunoprecipitation (ChIP) assays with polyclonal anti-Meq antibody. MSB-1 cells were grown in Leibowitz’s L-15 and McCoy 5A media supplemented with fetal bovine serum (15%), penicillin (100 IU/mL) at 37°C. Cells (10^7^) were cross-linked with formaldehyde (1%, 10 min, 37°C), which was added directly to the culture medium. The culture medium was removed and washed twice with ice cold phosphate-buffer saline (PBS) containing protease inhibitor cocktail (Sigma-Aldrich, P8340). ChIP was done using the Chromatin Immunoprecipitation Assay kit (Millipore, 17–295) exactly following manufacturer’s recommendations. Immunoprecipitation was performed with anti-Meq polyclonal antibody (1:100), incubated overnight at 4 °C. The DNA/Meq/antibody complexes were purified using Protein A-agarose/salmon sperm DNA beads. The purified complex sample was reverse cross-linked separating the DNA from Meq and its interacting proteins. Proteins that were co-immunoprecipitated with Meq were analyzed and identified by 2D LC ESI MS/MS as described above.

### Plasmid construction

The CD30 promoters (2431–2438 bp upstream from the ATG start codon) of six different chicken lines (6_1_, 7_1_, 7_2_, 15I_5_, N and P) were amplified by PCR with Pfu polymerase (Promega) and primers CD30-F and CD30-R. The amplified promoters were ligated into pCR®2.1-TOPO® (Life Technologies) producing pCR®2.1-CD30 plasmids. The cytomegalovirus (CMV) promoter in the pd2EGFP-N1 plasmid (Takara Bio) was removed by digestion with XhoI and VspI; linear DNA was blunt-ended by T4 DNA polymerase and then self-ligated producing pd2EGFPΔCMV. CD30 promoters were released from the pCR®2.1-CD30 plasmids by EcoRI digestion and ligated into EcoRI-linearized pd2EGFPΔCMV resulting in production of the six new expression plasmids pd2EGFP-CD30. The Meq promoter (1 kb upstream from the Meq start) of the virulent MDV-1 strain RB-1B was amplified by PCR with primers MEQ-F and MEQ-R. The promoter was first cloned into pCR®2.1-TOPO®, then released by EcoRI digestion and re-cloned into EcoRI-linearized pd2EGFPΔCMV producing the reporter plasmid pd2EGFP-Meq. The chicken cDNA encoding the NF-κB p100 [140] was released from the cloning vector pBS KS(+) with HindIII and XbaI and inserted into HindIII and XbaI-linearized expression vector pBK-CMV (Agilent Technologies), resulting in pBK-CMV-p100. The cDNA encoding the chicken NF-κB p105 [141] cloned in pGEM4 was released by digestion with EcoRI and KpnI and inserted into EcoRI- and KpnI-linearized pBK-CMV, producing pBK-CMV-p105. The ankyrin repeats were removed from the 5’ end of the NF-κB p105 cDNA by digestion with SacI. The chicken NF-κB p65 cDNA cloned in pTZ18R was released by digestion with *Xho*I and *Mfe*I and re-cloned into *Xho*I and *Sma*I-linearized pBK-CMV producing pBK-CMV-p65. Plasmids were purified using the affinity chromatography columns (Qiagen) and proper structure of all the plasmids was verified by restriction enzymes digest and sequencing.

### Promoter assays

The activity of CD30 and Meq promoters was analyzed in vitro by promoter reporter assays. First, the reporter gene d2EGFP was placed under the control of the CD30 and Meq promoters and the coding sequences of transcription factors (Meq oncogene, and NF-κB p65, p100 and p105) were cloned into the expression plasmid pBK-CMV (see Construction of plasmids above). The promoter reporter plasmids and transcription factor expression plasmids were then transfected into SOgE cells [101], and the expression of the reporter gene was quantitatively measured by duplex real-time PCR as described below.

SOgE cells were grown in Dulbecco’s modified Eagle’s minimum essential medium supplemented with 10% fetal calf serum, penicillin (100 IU/mL), streptomycin (100 mg/mL) and amphotericin B (0.25 μg/mL) at 37°C with 5% CO_2_. Plasmids were transfected in triplicate into SOgE cells in 24 well plates at 80% confluence using Lipofectin® reagent (Life Technologies) following the manufacturer’s instructions. Each well was transfected with 200–400 ng of DNA. To determine the effect of the Meq oncogene on the activity of the chicken CD30 promoters SOgE cells were transfected with either pUC18 alone (200 ng/well, negative control), pd2EGFP-N1 alone (200 ng/well, positive control), pd2EGFP-CD30 alone (200 ng/well, to measure the baseline level of expression from each of the CD30 promoters), or with a mix of pBK-CMV-Meq and pd2EGFP-CD30 (200 ng of each plasmid/well). To determine the transactivation effect of the NF-κB transcription factors alone or in combination with the Meq oncoprotein on the Meq promoter SOgE cells were transfected with plasmid mixtures and DNA. Plasmid pUC18 was added to transfection mixtures to give total amount of 400 ng plasmid DNA per well whenever it was necessary. Total RNA was isolated from transfected SOgE cells 48 h post transfection using TRI reagent (Molecular Research Center) following the manufacturer’s instructions. Isolated RNA was treated with DNaseI, extracted with phenol/chloroform, precipitated with ethanol and resuspended in water.

The d2EGFP mRNA levels in transfected SOgE cells were quantified using the Platinum Quantitative RT-PCR ThermoScript One-Step System (Life Technologies). Both, d2EGFP and 28S rRNA amplicons, were designed using Beacon Designer (PREMIER Biosoft). The reaction mixture consisted of 2X ThermoScript Reaction buffer, 10 μM of each primer, 1 μM each of probes, Platinum *Taq* DNA polymerase and 1 μL of total RNA (5 ng/μL) and the total volume was made to 12.5 μL with RNAase free water as filler. Amplification and detection was done on iCycler iQ Real-Time PCR Detection System (Bio-Rad) with the cycle profile of 50°C for 30 min and 95°C for 5 min, followed by 45 cycles of 95°C for 15 s and 60°C for 1 min. Each QPCR experiment included, samples (in triplicate), two no-template controls and a dilution series (5, 10, 50, 100, 500 and 1000) of total RNA made by mixing a 10 μL aliquot from all samples). Standard curves for d2EGFP and 28S rRNA were generated from the dilution series and the ratio of coefficient of regression values (for 28S RNA and d2EGFP) was used to calculate correction factor for PCR efficiency between these two genes. Both d2EGFP and 28S rRNA cycle threshold (Ct) values were subsequently normalized for correction factor for PCR efficiency. Mean Ct value for 28S rRNA was used to normalize the d2EGFP Ct values for any volume error. The means of the normalized Ct values were used to compare the relative percent expression compared to d2EGFP expression driven by the CMV promoter by doing one way ANOVA.

### Gene ontology (GO) based phenotype modeling

GO was used to identify the phenotype of CD30^hi^ and CD30^lo^ cells, specifically with respect to GO terms which are associated with cancer [3]. The GO annotations were obtained using tools available at AgBase [142] and modeled as described previously in [3]. Briefly, all the annotations those were either agonistic or antagonistic to specific biological processes which included activation, angiogenesis, apoptosis, cell cycle, differentiation, DNA damage response, migration, oxidative stress, and proliferation and telomere maintenance [3], were selected and the difference between the number of agonistic and antagonistic annotations indicated the overall phenotype for that particular GO term. *GO-modeler* based modeling for T-regulatory cells was done as described in [6,12] for both transcriptomics and proteomics data.

### mRNA and protein expression comparison

We calculated the fold change in amount of mRNAs and proteins transcripts in CD30^hi^ cells compared to CD30^lo^ cells in semi-quantitative manner. For microarray data we calculated the fold change in terms of ratio of normalized fluorescent intensities; for proteomics data, fold change was calculated by taking the ratio of mean sum of XCorr of that protein in CD30^hi^ to CD30^lo^ cells.

## Competing interests

The authors declare that they have no competing interests.

## Authors’ contributions

JB did chicken infection experiment, RNA and protein isolation. SK and JB did protein isolation. SK did microarray, proteomics, and systems biology data analysis and wrote the manuscript. DK did plasmid construction, and transcription reporter assays and SNP assays. HI did the microarray experiments and data analysis. KP did the proteomics 2D LC ESI MS/MS experiment and data analysis. SS did ChIP assay. SB conceived the project, did data analysis and wrote the manuscript. HC and HZ provided the necessary support for microarray, western blot and ChIP experiments and reviewed the manuscript. All authors read and approved the final manuscript.

## Supplementary Material

Additional file 1 **Additional file 1 contains four worksheets, sheet 1 (CD30**^**hi**^**Vs. CD30**^**lo**^**protein expression): contains proteins identified from CD30**^**lo**^**and CD30**^**hi**^**cells, worksheet 2 (ChIP identified proteins): contains proteins identified in ChIP experiment.** Worksheet 3 (NF κB isoforms subcellular locations): contains actual numbers of peptides of different isoforms of NF-κB in nuclear and extranuclear fractions, and worksheet 4 (Phosphorylated peptides locations) contains actual numbers of phosphorylated and non-phosphorylated peptides identified. (XLS 1901 kb) Click here for file

Additional file 2 **Additional file 2 contains three worksheets, sheet 1 contains the results from the statistical analysis of microarray data, sheet 2 contains expression data for only miRNAs and sheet 3 contains predicted targets genes and the relative protein expression of the targets of the miRNAs.** (XLS 8422 kb) Click here for file

Additional file 3 **Additional file 3 contains mRNA and protein expression fold changes in terms of CD30**^**hi**^**over CD30**^**lo**^**cells.** (XLS 859 kb) Click here for file

Additional file 4 **Additional file 4 contains pentile wise Meq binding sites for 88 concordantly expressed host host gene products.** (XLS 30 kb) Click here for file
